# Is There Release from Masking from Isomorphism between Perception and Action?

**DOI:** 10.3390/brainsci4020220

**Published:** 2014-03-26

**Authors:** Tara C. Dennehy, Shanna Cooper, Tanaz Molapour, Ezequiel Morsella

**Affiliations:** 1 Department of Psychology, University of Massachusetts, Amherst, MA 01003, USA; E-Mail: tdennehy@psych.umass.edu; 2 Department of Psychology, Temple University, Philadelphia, PA 19122, USA; E-Mail: shanna.cooper@temple.edu; 3 Department of Clinical Neuroscience, Karolinska Institute, Stockholm SE-17177, Sweden; E-Mail: tanaz.molapour@ki.se; 4 Department of Psychology, San Francisco State University, 1600 Holloway Avenue, EP 301, San Francisco, CA 94132, USA; 5 Department of Neurology, University of California, 350 Parnassus Ave, Room 706, Box 1207, San Francisco, CA 94117, USA

**Keywords:** consciousness, ideomotor theory, perceptual resonance, perception-and-action

## Abstract

The phenomenon of “entry into awareness” is one of the most challenging puzzles in neuroscience. Research has shown how entry is influenced by processes that are “bottom-up” (e.g., stimulus salience, motion, novelty, incentive and emotional quality) and associated with working memory. Although consciousness is intimately related to action, action-based entry remains under-explored. We review research showing that action-related processing influences the nature of percepts already in conscious awareness and present three experiments that, using a “release-from-masking” technique, examine whether action plans can also influence that which enters awareness in the first place. The present data, though intriguing and consistent with previous research, are not definitive. The limitations and theoretical implications of the findings are discussed. We hope that these experiments will spur further investigation of this understudied topic.

## 1. Introduction

What renders an otherwise unconscious perceptual representation conscious? This phenomenon of “entry into awareness” (“entry”, for short; [1,2]) is one of the most challenging puzzles in neuroscience and science more generally [3,4]. Some research has illuminated how entry is influenced by processes that are “bottom-up” (e.g., stimulus salience, motion, novelty, incentive and emotional quality, *etc.*; [5]), attentional (e.g., [6,7,8]) or associated with the cognitive demands of future tasks [9]. Recent research has also shown that entry occurs more quickly for visual stimuli that match items held in working memory than for stimuli that do not match items held in working memory [10]. In these experiments [10], items were rendered subliminal through the technique of continuous flash suppression, in which a stimulus presented to one eye is rendered subliminal by presenting a dynamic visual pattern to the other eye [10]. In summary, a variety of research has examined how perceptual attributes, bottom-up factors and working memory-related processes can influence entry.

However, though consciousness is intimately related to action [11,12,13,14,15,16], a treatment of action-based entry is almost entirely absent in the literature, perhaps because action itself is an understudied phenomenon [17,18]. One benefit of an action-based approach is that in such an approach, there is less likelihood of conflating conscious and attentional processes [13], a recurring problem in consciousness research focused on perception (see [19]) or working memory.

### 1.1. Ideomotor Theory and Perceptual Resonance

What are current ideas regarding how action may influence entry? Many of the contemporary ideas regarding how action may influence entry stem from ideomotor theory (e.g., [20,21]). When popularizing this theory [22,23], William James [16] proposed that the mere thoughts of actions produce impulses that, if not curbed or controlled by thoughts of incompatible actions, result in the performance of the imagined actions (e.g., [20]). From this standpoint, activating representations of the perceptual effects of an action (e.g., a finger flexing) leads to the corresponding action, effortlessly and without awareness of the motor programs involved [24,25,26]. It is through such a mechanism that voluntary action can be guided through consciously-mediated processing.

According to recent versions of ideomotor theory [20,21], not only can the perceptual-like representations of action effects influence action, but action plans themselves can influence perceptual processing (such “common code” accounts of perception-and-action propose that this is so because perception and action share the same representational format [20]). For example, experiments based on ideomotor theory reveal that performing a clockwise turning motion of the hand while looking at a drawing of a pixelated circle can induce the illusion that the circle is rotating in a clockwise fashion [27,28]. This interaction between action and conscious perception has been called *perceptual resonance* [27]. Perceptual resonance reveals that action-related processing can influence the nature of objects that are already perceptually conscious, as when interpretation of an ambiguous object (e.g., a pixelated circle) is biased by ongoing action. Importantly, perceptual resonance depends to some extent on whether the visual stimuli and actions are expressed on the same plane [28]. For example, perceptual stimuli presented on the vertical plane (as occurs on a computer screen) are more likely to be influenced by action expressed on a similar plane (e.g., a chalkboard) than a different plane (e.g., a computer mouse pad).

Another recent finding demonstrates that action-related processing can influence not only what is already in consciousness, but also entry itself. The finding stems from an experiment involving binocular rivalry. In this paradigm, subjects are first trained to respond in certain ways when presented with certain visual stimuli (e.g., to press a button when presented with the image of a house). After training, a different stimulus is presented to each eye (e.g., an image of a house to one eye and of a tree to the other). Surprisingly, the subject does not consciously perceive both objects (e.g., a tree overlapping a house), but instead perceives the objects to alternate (e.g., a house followed by a tree), with only one object consciously perceptible at a time. During rivalry, the conscious percept is said to be “dominant”, and the unconscious percept is said to be “suppressed.”

In a variation of this paradigm, Maruya *et al.* [29] demonstrated that voluntary action can influence which percept enters awareness: The object that moved in synchrony with the participant’s voluntary movements of a computer mouse was dominant for a longer period of time and suppressed for a shorter period of time. Rivalry stimuli consisted of a radial grating (resembling the pattern on a dart board) and a rotating sphere that was transparent and defined solely by dots. Prior to the test, participants learned to move a computer mouse in a continuous left-to-right motion. Participants later performed this motion under conditions of rivalry. Maruya *et* *al.* [29] conclude, “conflict between two incompatible visual stimuli tends to be resolved in favor of a stimulus that is under motor control of the observer viewing that stimulus” (p. 1096), revealing “a strong link between action and perception” (p. 1090). This finding by Maruya *et al.* [29] is in line with the aforementioned finding of Wohlschläger [28], who reported that participants, while perceiving a perceptually bistable apparent rotation of an object, were more likely to perceive the object as rotating in the direction in which they happened to be rotating a knob [30]. Consistent with the finding by Maruya *et al*. [29], Doesburg, Green, McDonald and Ward [31] found in a psychophysiological study that it is only when a percept is dominant that perceptual processing associated with the percept is coupled with motor-related processes in frontal cortex. Accordingly, an electrophysiological study demonstrates that entry of any kind may require a top-down signal from frontal cortex [32]. (Additional evidence regarding entry that is consistent with ideomotor theory stems from research on sensory attenuation; see [33]).

It remains unclear why action-based processes show these effects in some circumstances, but not others [27]. Based on ideomotor theory, the *sensorium hypothesis* [26,34,35,36] predicts that, because action/motor processes are largely unconscious [24,26,37], entry should be influenced most by perceptual-based (and not action-based) events and processes (e.g., priming by perceptual representations) (see the brain stimulation evidence in [38,39]). Hence, few entry effects should arise from what can be construed as “pure” action-related processes (should there be such a thing; *cf.*, [20]). Thus, entry from action in Maruya *et al.* [29] may actually be the result of the more “perceptual” aspects of action production, such as perceptual-like action effect representations (or “Effektbild”; [22]) or “corollary discharges” from action plans [26,40,41]).

### 1.2. Strong Dimensional Overlap

From this standpoint, perception and action are intimately related; moreover, they may even share the same representational format, as in common code models of perception-and-action [20]. Entry is most influenced by what has traditionally been regarded as the perceptual end of the perception-action cycle [26,42]. This is evident when it comes to phenomenology. Research by Wohlschläger and other ideomotor theorists (e.g., [20]) suggests that action-based effects on awareness, such as perceptual resonance, require not only perturbation of the sensorium, but dimensional overlap (e.g., shared spatial dimensions) between actions and percepts (e.g., [27,43]). (For brevity’s sake, we subsume the terms “isomorphic” and “analogical” under the more general term *strong dimensional overlap*; based on Wohlschläger [28]. This notion is supported by the finding that perceptuosemantic representations can be activated by actions (e.g., arm movements) that share spatial features with those representations; [44]).

From this standpoint, we hypothesize that strong dimensional overlap in the sensorium between (a) perceptual representations and (b) action-based afference or re-afference influences action-based entry. Actions devoid of such overlap should yield negligible effects on the entry of perceptual representations into consciousness. At this stage of understanding, we also propose the following, parsimonious hypothesis. The action-related mechanisms that influence the nature of perceptual representations that are already in consciousness are the very same mechanisms that influence which perceptual representations enter consciousness in the first place.

## 2. Experimental Section

To begin to provide additional evidence for the strong dimensional overlap hypothesis, we conducted a series of experiments to evaluate whether action-based entry occurs when the action and percept share strong dimensional overlap. Our primary aim was to assess whether a subliminal stimulus (e.g., a nonsense object resembling the number “8”) would become consciously perceptible when participants continuously emit an action resembling the stimulus (e.g., continuously drawing a figure 8 on a computer tablet)*.* Each experiment used a stronger dimensional overlap manipulation than the previous experiment. In Experiment 1, we contrasted the entry effects of an action-based manipulation having no dimensional overlap and of a bottom-up perceptual cue. In Experiment 2, we examined the potential entry effects of weak dimensional overlap (action and percepts were isomorphic, but on different planes), whereas in Experiment 3, we examined such effects from strong overlap (action and percepts were isomorphic and on the same plane).

We predicted that strong dimensional overlap (and bottom-up perceptual cueing) would lead to entry effects. Our project is different from that of Wohlschläger [28] in that the perceptual object was not already in consciousness. It is also different from that of Maruya *et* *al.* [29], because the stimuli were subliminal and, thus, could not enter consciousness spontaneously, as occurs in binocular rivalry [45]. It is important to emphasize that we expected only weak experimental effects, because we are speaking not of modulating the nature of an object that is already in consciousness, but of the rare event of having something that should be subliminal enter into consciousness as a function of an experimental manipulation. Such “release from masking” is difficult and rare to instantiate [46]. Because the effects were expected to be so weak, measures were taken to increase the sensitivity of the paradigm. These measures are described below.

In all experiments, our stimuli were rendered subliminal through the technique of backward masking (discussed below). We carefully examined various techniques that render a stimulus subliminal. These techniques included binocular rivalry, inattentional blindness [47], flash suppression [48], the attentional blink [8] and object substitution masking [1]. For several reasons, we selected the more basic technique of backward masking (“masking” for short). First, the technique is very effective at rendering visual stimuli imperceptible. Second, we had used it successfully in many preliminary studies [47]. Third, masking has fewer shortcomings than the other techniques. For example, masking works successfully for almost every participant, while inattentional blindness fails to occur for roughly one-third of participants [47]. Fourth, as the standard paradigm for disrupting “re-entrant” processing [1], masking is easy to replicate with basic laboratory equipment and permits the experimenter to control exactly when a stimulus should be subliminal. No such control can be exerted in many of the other subliminal techniques.

**Figure 1:**
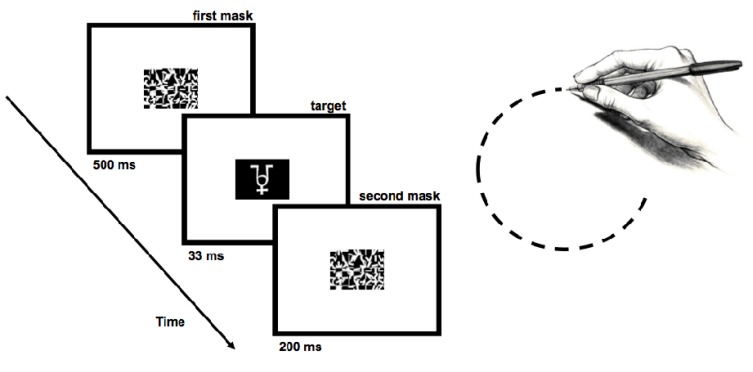
Schematic illustration of the time course of trial events in Experiment 1 (**left**). The illustration on the **right** side of the figure depicts the circular motion performed by participants in Experiment 2.

To instantiate masking in our experiments, we followed closely the procedures of previous studies [49,50]. Specifically, in our experiment, stimuli were rendered subliminal by presenting the targets (nonsense figures; Figure 1, left) momentarily (duration details presented below), with a pattern mask (taken from [49]) appearing briefly before and after the stimulus. Because action-based entry is such a rare event (especially when the targets are backward masked), we designed the following studies to be as sensitive as possible. To this end, we increased the likelihood of entry by presenting targets on each and every trial.

### 2.1. Experiment 1: No Dimensional Overlap *versus* Bottom-Up Salience

Several ideomotor-like approaches suggest that effects should stem not from any action-related processes, but only from the strength of perception-related processing (e.g., [26,51,52]). Hence, in Experiment 1, we compared the effects of an action-based manipulation without dimensional overlap with that of a bottom-up perceptual salience manipulation (“salience”, for short), in which the target is immediately preceded by a salient cue (e.g., a bright red frame). This manipulation was inspired by research showing that perceptual/attentional cueing can “release” stimuli from masking and increase the likelihood of entry (e.g., [2]).

Thus, in Experiment 1, the bottom-up signal from the target was strengthened by having a bright red frame appear briefly (500 ms) before target onset, surrounding the area where the target would later appear (e.g., [53]). It may well be that, as predicted (e.g., [26]), entry is a function primarily of bottom-up, perceptual processing. Establishing this would be an important theoretical advancement in its own right.

#### 2.1.1. Participants

San Francisco State University undergraduates (*n* = 53) participated for class credit.

#### 2.1.2. Stimuli

**Figure 2:**
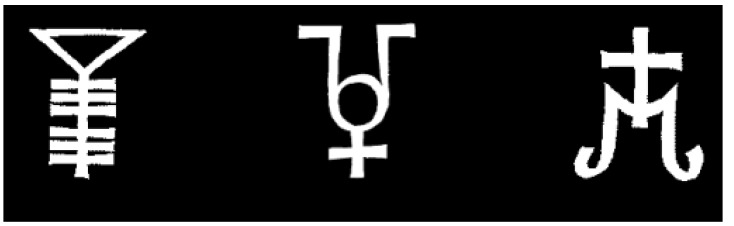
Visual targets used in Experiment 1.

Targets were three nonsense stimuli from Pessiglione *et* *al*. ([49]; Figure 2). We purposefully employed nonsense stimuli to avoid semantic labeling of the stimuli by participants. The pre- and post-masks (also from Pessiglione *et* *al.* [49]) consisted of fragments of the targets re-assembled randomly to create a composite pattern mask (Figure 1, left). Stimuli were presented via PsyScope software [54] on a 50.8 Apple iMac computer.

#### 2.1.3. Procedures

Prior to testing, participants were familiarized with the experimental stimuli (e.g., “The image below is a pattern mask, and it is NOT a target. It is important that you remember that pattern masks are not the same thing as targets.”). Participants completed two blocks of 45 trials in which, on each trial, one of the three targets was presented subliminally. Individual targets were randomly presented. During each trial, there was the presentation of a pattern mask (500 ms), followed by one of the targets (33 ms), which was then followed by a different pattern mask (200 ms; Figure 1, left). On one-third of the trials, the first pattern mask presented per trial appeared along with a salient cue: a red square surrounding the mask for 500 ms (following [49]). This was the cue (salience) condition. Per participant, the cue was always paired with only one of the three targets. Object-cue pairings were counterbalanced across participants. For 27 participants, the cue elicited an action: a button-press (action condition); the other 27 participants viewed the red square and did not respond to it (passive view condition).

Based on the procedures of [47], at the conclusion of each masking trial, participants inputted answers to questions about the subliminally-presented stimulus. Because piloting revealed that participants could perceive portions of otherwise subliminal stimuli, open-ended responses to one question were submitted to a content analysis in which independent judges (who were blind to condition) deliberated about which stimulus participants were most likely describing. Participants responded to the following fixed sequence of questions. (1) Did you see anything on the screen other than the pattern mask? (2) If YES, what do you think you saw? (If NO, enter N/A). (3) How confident are you that you saw something other than the pattern mask? (From 1 to 8, with 1 being not confident and 8 being very confident). (4) Did you see any of the following targets? (At this time, the three targets were presented again in a randomized array). If YES, please indicate which. If you do not think you saw a target, please guess. To avoid any effects stemming from the order in which three targets were presented, the spatial order of each target was varied randomly. We took care to verify that participants were not reporting the presence of the red square or the pattern mask when answering the questions after each trial.

To assess any mere exposure effects, in which target liking would increase as a function of conscious exposure to the stimuli [55], participants filled out a paper and pencil questionnaire following the computer-based portion of the study. The questions asked participants to rank the targets from “most liked” to “least liked.” Participants rated each of the targets individually on a scale from 1 to 8, with 1 being do not like and 8 being like a lot. Following the procedures of [56], at the conclusion of the study, a funneled debriefing form was administered to participants to assess their knowledge of the experimental manipulations and learn about their strategies for performing the task. The questions included, (1) What did you think the purpose of this experiment was? (2) What did you think this experiment was trying to study? (3) Why do you think you performed an action on some trials (pushing a button in response to a red square) and not on others? (4) Do you think the action you performed (pushing the button) was somehow related to what you experienced? (Questions 3 and 4 were presented only to participants in the action condition. Participants in the passive view condition responded instead to the following question: Did seeing the red square relate to how you perceived anything in the experiment?) (5) On what did you usually base your liking judgments (of the targets)? (6) Did you have any goal or strategy in completing the experiment? (7) What do you think the red square was for? Analysis of the debriefing data, including the target likeability judgments, revealed no systematic effects. Importantly, no participants discerned the hypotheses at hand.

### 2.2. Experiment 2: Weak Dimensional Overlap

In Experiment 1, it is possible that there was no effect of action on entry, because of the lack of dimensional overlap. In the weak dimensional overlap condition of Experiment 2, the action (*i.e.*, drawing a shape) and perceptual input (*i.e.*, a nonsense object resembling the shape) occurred on different planes (e.g., horizontal and vertical, respectively). After commencing the trial by pressing the space bar with the left hand, participants continuously performed an action with their right hand (e.g., drawing a circular figure on a computer tablet) that was spatially isomorphic to one of the nonsense figures (e.g., one containing a circle; Figure 1, right). To ensure that participants had enough time to perform the prescribed action continuously, the fixation following the ready prompt lasted 1500 ms.

We selected actions that could be expressed continuously, because, unlike button-pressing, they do not have a discrete beginning or end. Hence, they could be emitted continuously throughout the duration of the trial. So that all participants performed the action in a consistent manner, there was a brief training session prior to the test, to teach participants how to perform the prescribed drawing actions. During training, the experimenter demonstrated the correct motion and shape, but to prevent explicit semantic labeling, did not name the shape that was being drawn. During the test, a screen prevented participants from viewing the execution of their own actions, to eliminate spatial priming from vision.

#### 2.2.1. Participants

San Francisco State University undergraduates (*n* = 19) participated for course credit.

#### 2.2.2. Procedures

Participants were run individually in a within-subjects design. All the procedures were identical to those of Experiment 1, except for the following. Participants utilized a, unbeknownst to them, de-activated tablet-and-stylus (Wacom, Vancouver, WA, USA) to perform experimental motions. The tablet was placed to the right of the keyboard on the desk at which the participant was sat. Participants interacted with the tablet in a similar manner as they would interact with a mouse. An opaque partition was placed between the tablet and the computer screen so that participants could not see their own motion and be influenced by the visual input. To avoid any potential discomfort, participants were also given a cushion (*i.e.*, mouse pad) to place under their elbow. Importantly, the mouse pad rested on a horizontal plane, and stimuli were presented on a vertical plane, which decreased the overlap between perception and action.

During training, the experimenter also demonstrated experimental motions (resembling a circle or an upside-down triangle) that would be performed on the tablet. Experimental motions were blocked and counterbalanced across all participants. To diminish the effects of explicit semantic labeling, the experimenter demonstrated the motion, but never uttered the name of the motion being performed. To assess if participants understood the instructions, participants were then asked to demonstrate the motion on the tablet. Following training, participants completed two blocks of 45 trials in which, on each trial, one of the three targets was presented subliminally for 50 ms (the stimulus interval was increased due to the low proportion of no-cue trials on which participants identified seeing something in Experiment 1). Individual targets were randomly presented. The pairings of actions to targets were counterbalanced evenly across all participants. For each block, participants performed an action on the tablet with the stylus that was either isomorphic to, or not isomorphic to, the target. Action types were blocked and were fully counterbalanced across participants.

During each trial, participants were instructed to begin making the experimental motion on the tablet with the stylus. This was followed first by a pattern mask (200 ms), then by one of the targets (50 ms) and then by a different pattern mask (200 ms). After the second pattern mask, participants were instructed to stop making the experimental motion on the tablet with the stylus.

The funneled debriefing questions for this study included: (1) What did you think the purpose of this experiment was? (2) What did you think this experiment was trying to study? (3) Why do you think you performed a motion during the trials (tracing the shape on the tablet with the stylus)? (4) Do you think the motion that you performed was somehow related to what you experienced? (5) On what did you usually base your liking judgments (of the targets)? (6) Did you have any goal or strategy in completing the experiment? Analysis of the debriefing data revealed that no participants discerned the hypotheses at hand.

### 2.3. Experiment 3: Strong Dimensional Overlap

**Figure 3:**
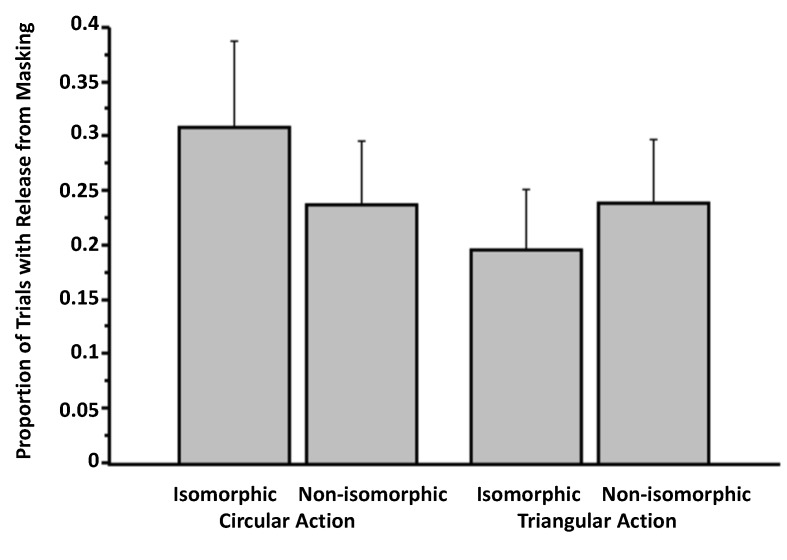
Proportion of trials involving release from masking as a function of action and isomorphism (Experiment 2). The data are culled from the question, “Did you see anything on the screen other than the pattern mask?” Error bars signify SEMs.

Perhaps our circular and triangular actions constituting our weak overlap manipulation did not influence entry, because the action and visual target were not sufficiently isomorphic. For instance, in our experimental set-up, the actions were expressed on a tablet resting horizontally on the surface of a table, and the visual stimuli were presented on the vertical surface of the computer screen. Whether the visual stimuli and actions are expressed on the same plane are important for perceptual resonance ([28]). We examine this possibility in Experiment 3, in which visual targets and the drawing actions are both expressed on the same vertical plane. We also took the opportunity to improve other aspects of our experimental arrangement. Experiment 3 was also motivated by the presence of some statistical trends and by inspection of the pattern in Figure 3.

Experiment 3 followed the general procedures as those of Experiment 2, except that now there was strong dimensional overlap. In addition, the masked stimuli were different. Based on content analysis from Experiment 2 indicating that participants may have seen parts of the target stimuli, but were unable to identify enough of a given stimulus to be accurate, we used familiar, simple target shapes: a circle, triangle and plus sign). Two of these shapes (circle, triangle) were isomorphic to the motion participants made with their hand. The visual objects were of the same size as the targets of Experiment 2.

#### 2.3.1. Participants

San Francisco State University undergraduates (*n* = 34) participated for class credit.

#### 2.3.2. Procedures

Unlike in Experiment 2, when participants interacted with the tablet on a horizontal plane, participants in Experiment 3 interacted with the tablet, which was held by an iPad tablet stand (Brookstone, Merrimack, NH, USA), on a vertical plane at the same level as the stimuli presented on the computer screen, such that the participant interacted with the tablet the way one would interact with a chalkboard. This arrangement was motivated by research [28] suggesting perceptual resonance occurs best when action effects are performed on the same spatial plane as the percept. As in Experiment 2, an opaque partition was placed between the tablet and the computer screen so that participants could not see their own motion and be influenced by the visual input. The additional procedures, post-trial questions and funneled debriefing questions were identical to those of Experiment 2. No participants discerned the hypothesis at hand.

## 3. Results and Discussion

### 3.1. Experiment 1

**Figure 4:**
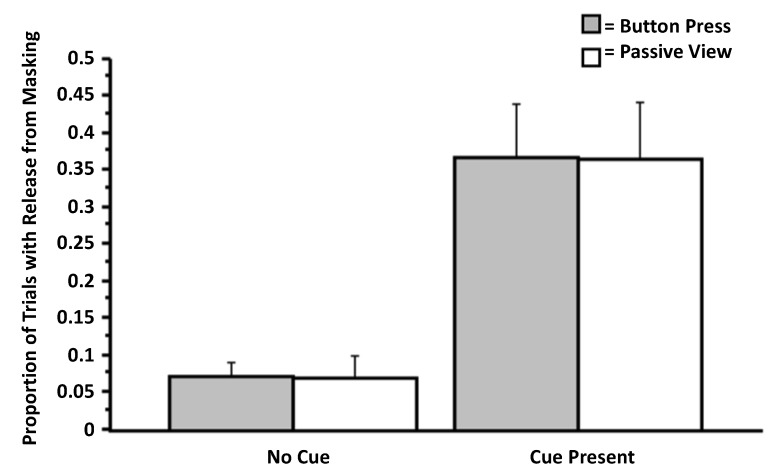
Proportion of trials involving release from masking as a function of bottom-up perceptual cueing and action (a simple button press) (Experiment 1). The data presented here stem from the question, “Did you see anything on the screen other than the pattern mask?” Error bars signify SEMs*.*

**Figure 5:**
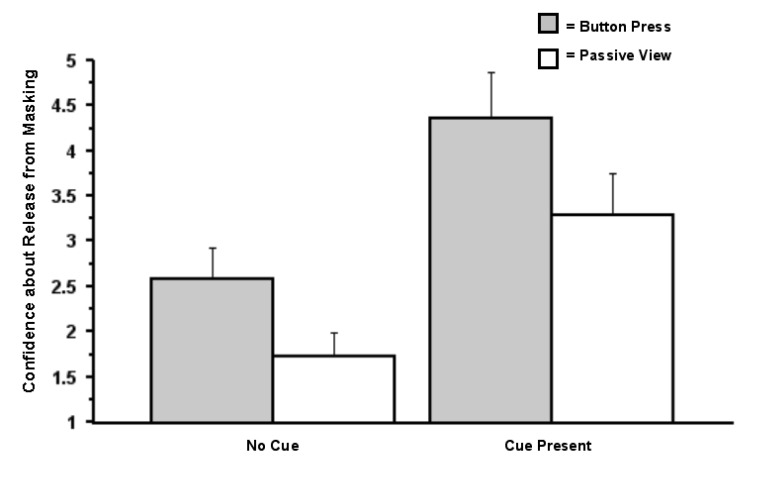
Confidence regarding judgments about release from masking (Experiment 1). The data presented here stem from the question, “How confident are you that you saw something other than the pattern mask?”

Notably, we found no effect of action on entry into conscious awareness. In an ANOVA on the data from Question 1 (“Did you see anything on the screen other than the pattern mask?”), there was no effect of action (button press *versus* passive view), *F*(1, 51) = 0.001, *p* = 0.98, but there was an effect of salience (cue present *versus* absent), *F*(1, 51) = 34.59, *p* < 0.0001 (η*_p_*^2^ = 0.99), and no interaction between the factors, *F*(1, 51) < 0.01, *p* = 0.99. It appears that, when the target was surrounded by the salient cue, information about targets was more likely to be released from masking than when no such cue was presented (Figure 4). Importantly, this analysis excluded cases in which the participant reported awareness of the mask or red square. Analysis of participants’ confidence about these judgments (Question 3: “How confident are you that you saw something other than the pattern mask?”) revealed a main effect of action, *F*(1, 51) = 4.18, *p* = 0.046 (η*_p_*^2^ = 0.08), a main effect of the presence of the red cue, *F*(1, 51) = 26.57, *p* < 0.0001 (η*_p_*^2^ = 0.99), and no interaction between the two factors, *F*(1, 51) = 0.099, *p* = 0.75 (η*_p_*^2^ = 0.002; Figure 5). Analysis on arcsine transformations of the proportion data revealed the same pattern of results (arcsine transformations are often used to statistically normalize data that are in the form of proportions).

Regarding the data in which participants were asked to identify the target (Question 4: “Did you see any of the following targets? If YES, please indicate which. If you do not think you saw a target, please guess”), there was no effect of action, *F*(1, 51) = 2.38, *p* = 0.13 (η*_p_*^2^ = 0.04), no effect of presentation of the red cue, *F*(1, 51) = 0.40, *p* = 0.53 (η*_p_*^2^ = 0.31), and no interaction between the two factors, *F*(1, 51) = 0.90, *p* = 0.35 (η*_p_*^2^ = 0.02). Again, analysis on arcsine transformations of the proportion data revealed the same pattern of results.

One must interpret the significant effect from the salience manipulation with caution: it may well be that the presence of the red cue led participants to conclude that, with the occurrence of such an event, something must have been presented. This possibility could lead to an artifact from the response bias on the part of the participants.

### 3.2. Experiment 2

With the weak overlap manipulation, we again found no evidence of release from visual masking from expressing an action plan that is isomorphic to the masked target (*i.e.*, the isomorphic condition). For example, when asked to select the masked target from an array of all three targets (in response to Question 4: “Did you see any of the following targets? If YES, please indicate which. If you do not think you saw a target, please guess”), the proportion of trials in which there was a correct identification was comparable in the isomorphic and control conditions. In our ANOVA on the proportion of trials in which there was the correct identification of the target (Question 4), action (circular *versus* triangular) was one within-subjects factor and isomorphism (isomorphic *versus* control) was another within-subjects factor. In this analysis, there was no main effect of action on correct identification of targets, *F*(1, 18) = 1.06, *p* = 0.32 (η*_p_*^2^ = 0.06), no main effect of isomorphism on the dependent measure, *F*(1, 18) = 0.26, *p* = 0.61 (η*_p_*^2^ = 0.01), and no interaction between the two factors, *F*(1, 18) = 2.09, *p* = 0.17 (η*_p_*^2^ = 0.10). A similar lack of contrast between the isomorphic and non-isomorphic condition was found in the data resulting from Question 1 (“Did you see anything on the screen other than the pattern mask?”). In an ANOVA, there was no main effect of action, *F*(1, 18) = 1.76, *p* = 0.20 (η*_p_*^2^ = 0.09), no main effect of isomorphism, *F*(1, 18) = 0.82, *p* = 0.38 (η*_p_*^2^ = 0.04), and only a trend of an interaction between the two factors, *F*(1, 18) = 2.92, *p* = 0.10 (η*_p_*^2^ = 0.14; Figure 3). Regarding participants’ confidence about these judgments (Question 3: “How confident are you that you saw something other than the pattern mask?”), there was only a trending main effect of action, *F*(1, 18) = 3.07, *p* = 0.10 (η*_p_*^2^ = 0.15), a marginal main effect of isomorphism, *F*(1, 18) = 4.08, *p* = 0.059 (η*_p_*^2^ = 0.18), and no interaction between the two factors, *F*(1, 18) = 2.38, *p* = 0.14 (η*_p_*^2^ = 0.62).

Analysis on arcsine transformations of all the proportion data revealed the same pattern of results. We took the opportunity to content analyze the free responses to Question 2 (“What do you think you saw?”), but found nothing that was significant or noteworthy. This outcome may be because the experiment was not designed to yield data for content analysis.

### 3.3. Experiment 3

**Figure 6:**
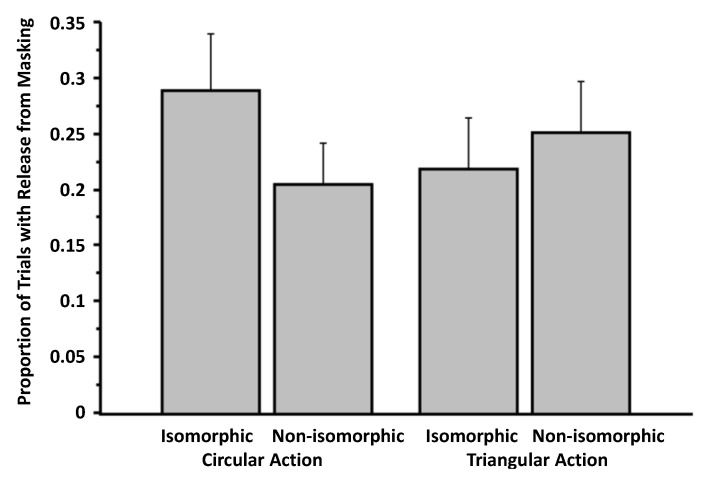
Proportion of trials involving release from masking as a function of action and isomorphism (Experiment 3). The data are culled from the question, “Did you see anything on the screen other than the pattern mask?” Error bars signify SEMs.

Although the results of this experiment are by no means definitive (for the reasons outlined below), the experiment provided some initial data that the action manipulation may have had some influence on the release of visual masking of targets. For example, examination of participants’ detection of something other than the pattern mask (*i.e.*, the information from Question 1) reveals an interesting interaction between action (circular *vs.* triangular) and isomorphism (isomorphic *vs.* non-isomorphic), *F*(1, 33) = 12.09, *p* = 0.001 (η*_p_*^2^ = 0.27), but no main effect of action, *F*(1, 33) = 0.27, *p* = 0.61 (η*_p_*^2^ = 0.01) and no main effect of isomorphism, *F*(1, 33) = 2.33, *p* = 0.14 (η*_p_*^2^ = 0.07; Figure 6). It seems that entry may have been influenced significantly only when participants performed the circular action. Consistent with this interpretation, the contrast between the two bars on the left of Figure 6 (*i.e.*, circular action: isomorphic *vs.* non-isomorphic) is significant, *t*(33) = 3.18, *p* = 0.003, but not that between the two rightmost bars, which reflect the condition involving the triangular action, *t*(33) = 1.54, *p* = 0.13. Accordingly, 20 of out of 34 subjects in the data analysis revealed higher entry rates for isomorphism (in the circle condition), and only six showed an effect in the unpredicted direction. Eight participants showed no effect. However, in the condition involving triangular action, 16 participants showed the opposite effect, and only eight showed an effect from isomorphism (ten participants showed no effect). It is possible that the circular motion may have been easier to sustain and perform continuously than the triangular motion.

**Figure 7:**
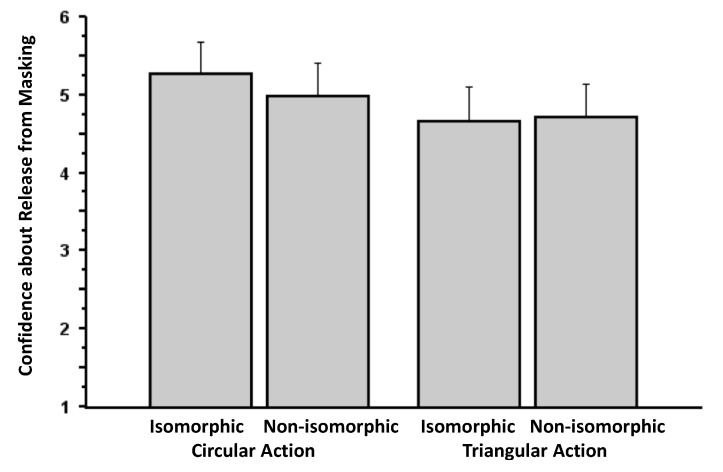
Confidence regarding judgments about release from masking (Experiment 3). The data presented here stem from the question, “How confident are you that you saw something other than the pattern mask?” Error bars signify SEMs.

Consistent with this interpretation of the interaction, analysis of participants’ confidence about these judgments revealed a main effect of action, *F*(1, 33) = 4.79, *p* = 0.036 (η*_p_*^2^ = 0.13), no main effect of isomorphism, *F*(1, 33) = 2.48, *p* = 0.12 (η*_p_*^2^ = 0.07), and a similar interaction between the two factors, *F*(1, 33) = 4.75, *p* = 0.037 (η*_p_*^2^ = 0.13; Figure 7). These unexpected interactions are worthy of future investigation.

Consistent with these two analyses, a content analysis on the responses that participants provided to the open-ended question about what they saw reveals that target-related verbal content was influenced by the experimental manipulation. The instructions to the judges were as follows. “In order to make your ratings, please study the images (triangle, circle, plus) below. Each participant has provided a description (ranging from a single character to several words) of what they felt they saw on trials where they indicated that they had seen something other than the pattern mask. Please note that in some cases, participants may have used a single letter or number to denote the shape they saw, as in “+”. As the target was masked, the descriptions may not be very accurate, but they may capture elements of the images below.” The three objects were presented below, with a letter next to each object. Judges indicated the letter associated with the object that was most like the description. Judges agreed in the majority of cases (99.77%; 3053/3060 total trials), as participants typically used the actual stimuli names (triangle, circle, plus) in the open-ended response section.

**Figure 8:**
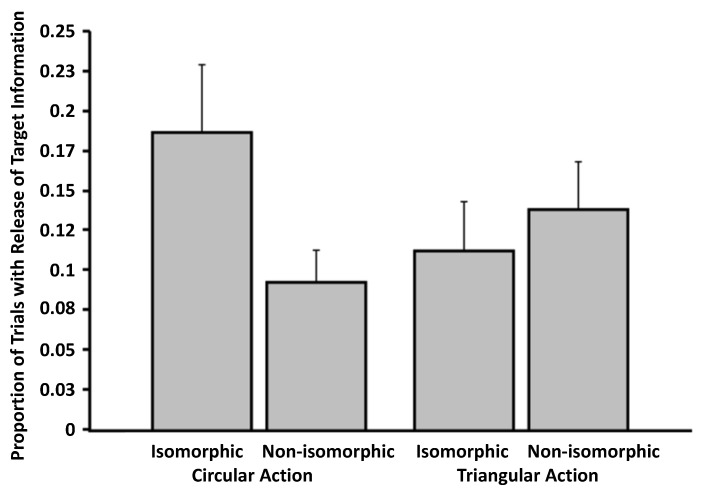
Proportion of trials involving release from masking as a function of action and isomorphism (Experiment 3). The data are culled from a content analysis of participants’ descriptions about targets. Error bars signify SEMs.

**Figure 9:**
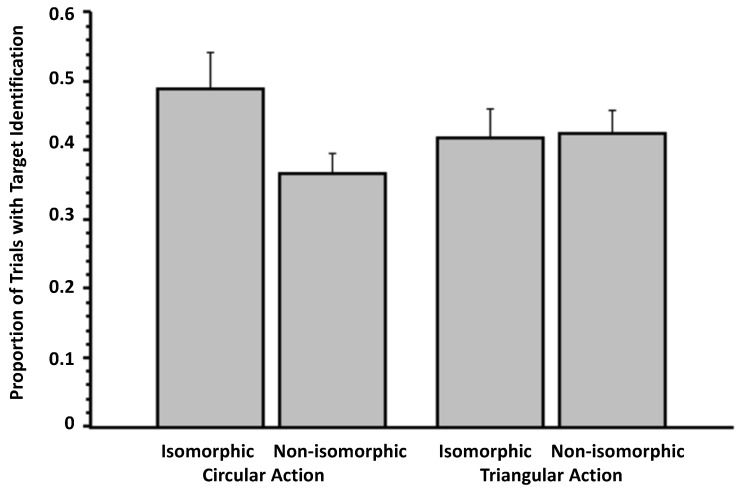
Proportion of trials in which participants correctly identified visual targets, as a function of action and isomorphism (Experiment 3). Data stem from the question, “Did you see any of the following targets? If YES, please indicate which one.” Error bars signify SEMs.

Analysis of the content data revealed an interaction pattern resembling that of Figure 6: an interaction between action and isomorphism, *F*(1, 33) = 11.54, *p* = 0.002 (η*_p_*^2^ = 0.26), a marginal main effect of isomorphism, *F*(1, 33) = 2.89, *p* = 0.098 (η*_p_*^2^ = 0.08), and no main effect of action, *F*(1, 33) = 0.56, *p* = 0.46 (η*_p_*^2^ = 0.02; Figure 8). However, it seems that our experimental manipulation did not lead to the correct selection of targets during the critical trials. In this analysis, there was no effect of action, *F*(1, 32) = 0.13, *p* = 0.72 (η*_p_*^2^ = 0.01), no main effect of isomorphism, *F*(1, 32) = 1.24, *p* = 0.27 (η*_p_*^2^ = 0.04), and only a marginal interaction between the two factors, *F*(1, 32) = 3.34, *p* = 0.077 (η*_p_*^2^ = 0.09; Figure 9). Repetition of all the proportion analyses with arcsine transformed versions of the data yield the same pattern of results.

## 4. Conclusions

Previous research suggests that action-based effects on conscious awareness (e.g., perceptual resonance) require not only perturbation of the sensorium, but strong dimensional overlap (e.g., shared spatial dimensions) between actions and percepts [20,27,28,43,44]. Research has shown that such overlap can influence the nature of percepts already in conscious awareness. Little research to date has examined whether dimensional overlap can influence that which enters conscious awareness in the first place (*cf.* Maruya*. et al.* [29]). It has been hypothesized that strong dimensional overlap in the sensorium (from action-based afference or re-afference) could influence entry and that action devoid of such overlap would yield negligible entry effects.

In our series of experiments, we found little evidence of action-based entry. Although there was some evidence in Experiment 3 that, consistent with previous research, actions having strong dimensional overlap with visual targets seem to have released some information about masked targets, this finding is the only significant effect of action-based entry among a group of marginal or null effects. This finding would thus require extensive replication before strong conclusions can be drawn. Our null effects may reflect insufficient statistical power. Regarding the null effects, because this area of research is uncharted territory and little is known about the nature of these kinds of effects, it is difficult to anticipate *a priori* what would constitute a meaningful effect size.

If future experiments replicate our significant effect, then the present finding might constitute a conceptual replication of Wohlschläger [28] with stimuli below the threshold of conscious awareness. If allotted the sufficient resources and session length, future investigations could attempt to include, in one experimental study, the three levels of isomorphism (no isomorphism, weak overlap and strong overlap) that we examined across our series of experiments. Constrained by several factors (e.g., the length of the experimental session), we were not able to conduct one experiment that included all levels of interference. Such an experiment would need to have the presentation of block order counterbalanced across subjects. One potential disadvantage of such an experiment would be that participants would be exposed to the subliminal stimuli for three blocks of trials. Such an extensive exposure to the subliminal stimuli could diminish the effectiveness of the masking manipulation.

As anticipated, our effects of action-based entry in Experiment 3 were weak and far from straightforward. For example, action-based entry influenced participants’ descriptions of targets, but did not influence the correct identification of targets. It is important to emphasize that, in the cases in which action might have influenced entry, it did not lead to correct identification of the targets. Instead, it led to the release of some information about the targets. In addition, though it remains unclear why, the effects of isomorphism were detectable for conditions involving circular actions, but not for those involving triangular actions. This unexpected interaction, which was also reflected in participants’ confidence judgments about reports, beckons future investigation. Perhaps our effect stemmed not from action-based processing, but from participants forming an iconic representation about the circular motion (e.g., [10]). Such a possibility, too, could be the subject of future investigation. Another limitation of our approach is that, to maximize the sensitivity of our experiments, targets were presented on every trial.

Consistent with the sensorium hypothesis, in Experiment 1, bottom-up perceptual cueing increased the probability of entry of otherwise subliminal targets, which is consistent with the findings of Mathewson *et al.* [2]. Again, this effect could have been an artifact of response bias from participants assuming that, with the occurrence of the salient cue, something must have been presented. Whether such a bias occurred, and the degree to which it occurred, would be easier to appreciate had our studies included trials in which no targets were presented. We urge future research investigating this possibility. Unfortunately, and as discussed above, we did not include such trials in the present research in order to maximize the sensitivity of our experimental paradigms. In contrast to the perceptual cue in Experiment 1, action production (a simple button press, with no dimensional overlap of targets) did not influence entry in any discernible way. From this manipulation, we also learned that participants often perceive only portions of targets, even when target identification fails. This led to the development of a dependent measure that was more amenable to content analysis, a more fine-grained measure than simple detection and/or identification. The observation that, in our effect, participants may have partial conscious access to the subliminally-presented stimulus has implications for current theorizing about consciousness [57,58]. Future variants of our paradigm could also examine whether sensory attenuation [59] or “blindness to response-compatible stimuli” [60] could arise from our manipulations. In the present studies, we did not have the conditions necessary for such effects to arise. Despite the above limitations, we hope that this report provides a foundation for, and spurs future research on, the understudied topic of action-based entry. Given the intimate relationship between action and awareness and because it is more experimentally tractable to study the relationship between action and awareness than the relationship between attention and awareness (the traditional approach; e.g., [19]), action-based entry provides a unique portal through which to unravel the nature of entry while also advancing theories on action and conscious awareness. We believe that an action-based approach would provide findings that would complement data from neuroscientific approaches focusing on the better-known, attentional and perceptual determinants of entry (e.g., [29]).
